# Biological optimization for mediastinal lymphoma radiotherapy – a preliminary study

**DOI:** 10.1080/0284186X.2020.1733654

**Published:** 2020-03-27

**Authors:** Laura Ann Rechner, Arezoo Modiri, Line Bjerregaard Stick, Maja V. Maraldo, Marianne C. Aznar, Stephanie R. Rice, Amit Sawant, Søren M. Bentzen, Ivan Richter Vogelius, Lena Specht

**Affiliations:** a Department of Oncology, Section of Radiotherapy, Rigshospitalet, University of Copenhagen, Copenhagen, Denmark;; b Niels Bohr Institute, Faculty of Science, University of Copenhagen, Copenhagen, Denmark;; c Department of Radiation Oncology, University of Maryland School of Medicine, Baltimore, MD, USA;; d Manchester Cancer Research Centre, Division of Cancer Sciences, The University of Manchester, Manchester, UK;; e Clinical Trial Service Unit, Nuffield Department of Population Health, University of Oxford, UK;; f University of Maryland Medical Center, Baltimore, MD, USA;; g Greenebaum Comprehensive Cancer Center, Department of Epidemiology and Public Health, University of Maryland School of Medicine, Baltimore, MD, USA

## Abstract

**Purpose:** In current radiotherapy (RT) planning and delivery, population-based dose-volume constraints are used to limit the risk of toxicity from incidental irradiation of organs at risks (OARs). However, weighing tradeoffs between target coverage and doses to OARs (or prioritizing different OARs) in a quantitative way for each patient is challenging. We introduce a novel RT planning approach for patients with mediastinal Hodgkin lymphoma (HL) that aims to maximize overall outcome for each patient by optimizing on tumor control and mortality from late effects simultaneously.

**Material and Methods:** We retrospectively analyzed 34 HL patients treated with conformal RT (3DCRT). We used published data to model recurrence and radiation-induced mortality from coronary heart disease and secondary lung and breast cancers. Patient-specific doses to the heart, lung, breast, and target were incorporated in the models as well as age, sex, and cardiac risk factors (CRFs). A preliminary plan of candidate beams was created for each patient in a commercial treatment planning system. From these candidate beams, outcome-optimized (O-OPT) plans for each patient were created with an in-house optimization code that minimized the individual risk of recurrence and mortality from late effects. O-OPT plans were compared to VMAT plans and clinical 3DCRT plans.

**Results:** O-OPT plans generally had the lowest risk, followed by the clinical 3DCRT plans, then the VMAT plans with the highest risk with median (maximum) total risk values of 4.9 (11.1), 5.1 (17.7), and 7.6 (20.3)%, respectively (no CRFs). Compared to clinical 3DCRT plans, O-OPT planning reduced the total risk by at least 1% for 9/34 cases assuming no CRFs and 11/34 cases assuming presence of CRFs.

**Conclusions:** We developed an individualized, outcome-optimized planning technique for HL. Some of the resulting plans were substantially different from clinical plans. The results varied depending on how risk models were defined or prioritized.

## Introduction

The prognosis for patients with Hodgkin lymphoma (HL) with modern combined modality therapy (CMT) is excellent for patients with early stage disease, with 5-year freedom from treatment failure over 85–90% [1,2], and still good for patients with advanced disease, with a 3-year progression free survival (PFS) of around 75% [3]. It is well known that radiation therapy to the mediastinum increases the risk of second malignancies [4,5], primarily in the breasts in female patients and in the lungs, and the risk of cardiac morbidity and mortality [6–9]. Though modern radiotherapy (RT) approaches such as involved site radiation therapy (ISRT) [10–13] lead to much lower doses and smaller fields than mantle field irradiation, which was abandoned 15–20 years ago, minimizing the risk of serious long-term effects of RT in each individual patient remains important.

The goal of RT planning is to optimize the therapeutic ratio, and population-based dose-volume constraints are used in current clinical practice to limit the risk of adverse effects. However, using population constraints may be unreasonable in an individual patient due to the patient’s anatomy relative to the target volume and patient-specific risk factors. For example, for patients with extensive disease, doses close to or exceeding the usual constraints may be accepted if maximum tumor control is prioritized. Conversely, for patients with limited disease, the conventional dose constraints may lead to acceptance of plans that are suboptimal. Ideally, doses to all normal structures should be kept as low as reasonably achievable, with emphasis on the most critical organs [14]. In the particular case of early-stage HL, it might even be clinically preferable in some cases to accept a small compromise of target coverage in order to reduce the dose to a critical OAR and the associated risk of late effects. However, most treatment planning systems are not suited to this scenario, and those compromises are performed subjectively. To this end, we need a flexible dose-planning tool that allows us to optimize the tradeoff between the risks of recurrence and various severe late effects to achieve the best overall outcome for each patient with HL by optimizing life expectancy.

To directly balance these tradeoffs during planning, the incorporation of biological or normal tissue complication probability (NTCP) models into treatment plan optimization has been proposed [15–19]. Various biological optimization methods have been explored for disease sites such as prostate cancer [20–23], head and neck cancer [24,25], brain cancer [26], breast cancer [27], and intrahepatic tumors [28]. For HL, a tool for evaluating biological endpoints for already created treatment plans has been developed [29], but the direct application of biological models in RT plan optimization, to the authors’ knowledge, has not yet been investigated. In addition, most reports focus on the probability of a side effect, without considering that the severity/mortality burden is greater from some than others (e.g., second lung cancer vs second breast cancer). The goal of this study was to explore the potential benefit of an individualized O-OPT HL RT planning approach which considers the dose-response effect of radiation on tumor control and mortality from late effects of treatment simultaneously. We also investigated the sensitivity of the O-OPT plan result to model parameters.

## Material and Methods

### Patients

CT datasets from 34 patients (17 male, 17 female) with biopsy proven mediastinal HL and median age 33.5 years (range 16–76), were selected for this retrospective study. All patients were treated between 2006 and 2010 (Figure S1 in the supplementary material) using the involved node radiation therapy approach (INRT) [30]. Contours from the clinical RT plans were used for O-OPT planning, and the clinical plans were used as a reference for comparison with O-OPT plans. The clinical plans were 3 D conformal radiation therapy (3DCRT), mostly with anterior-posterior posterior-anterior (AP-PA) beam setup with energy of 6 MV. In addition, 2-arc volumetric modulated arc therapy (VMAT) plans were also used for comparison with O-OPT plans [31]. All clinical 3DCRT and VMAT plans were renormalized to a mean dose of 30.6 Gy to the clinical target volume (CTV) (1.8 Gy/fraction) for this study (AAA, Eclipse V13.6 Varian Medial Systems, Palo Alto, CA).

### Preplanning beam setup

**Figure 1.**
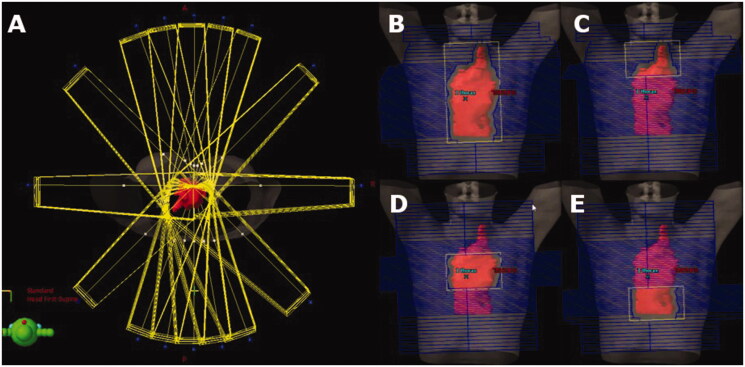
Beam setup for preplanning before optimization with 16 beam angles surrounding the patient (A) and examples of open and partially closed subfields that were created for each beam angle (B–E). The CTV is shown in red.

Preplanning was performed by creating a set of beams from which the optimization algorithm could choose for the O-OPT plans. Sixteen co-planar gantry angles (0, 10, 20, 45, 90, 135, 160, 170, 180, 190, 200, 225, 270, 315, 340 and 350 degrees) were considered in addition to the clinically used angles if not listed. The higher resolution of angles in the AP-PA regions was chosen to mimic a ‘butterfly’ technique as an option in the solution space [32]. The majority of fields were 6 MV, except two patients who were planned with 18 MV fields, following their 3DCRT clinical plans. To allow basic dose modulation, four fields were created for each angle: one open field and three subfields that together fully covered the target (e.g., superior, middle, and inferior), yielding a total of at least 64 beams (Figure 1). Initial doses from each beam were calculated using a commercial treatment planning system (TPS) (AAA, Eclipse V13.6). Equal field weighting was set in Eclipse TPS, which implies same dose contribution to isocenter from each field (therefore, differing monitor units). These dose matrices were exported for optimization.

### Inverse plan optimization

Inverse planning was performed by our in-house particle swarm optimization (PSO) engine, implemented in MATLAB (R2016a; MathWorks, Matick, MA, USA) [33]. PSO algorithm’s highly-parallelizable, metaheuristic and global nature has been thoroughly introduced in the literature [34,35] and applied to various RT inverse planning studies [36–38]. PSO searches the solution space with a group (swarm) of search agents, called particles, that communicate with each other while exploring the solution space individually. In this study, a swarm of 30 particles were used over 30 iterations, and a perturbation was applied at iteration 20 to avoid local minima. At each perturbation step, the particle with the best solution was kept, while the others were randomly re-distributed without erasing their personal bests from their memories. The output of the optimization was a list of monitor unit (MU) values for each beam that described the O-OPT plan. Beams with <5 MU were eliminated during optimization. If the optimization algorithm created a plan that was equal in risk with the clinical 3DCRT plan, the clinical 3DCRT plan was chosen. The objective function was defined as a summation of the probabilities of disease recurrence and the normal tissue complications given by the equation

(1) $$p_{\mathrm{tot}}=p_{\mathrm{DR}}+p_{\mathrm{CHD}}+p_{\mathrm{LC}}+p_{\mathrm{BC}}$$

where $p_{\mathrm{tot}}$ is the total penalty function, and the penalty functions for disease recurrence, mortality due to coronary heart disease, mortality due to secondary lung cancer, and mortality due to secondary breast cancer are given by $p_{\mathrm{DR}},\;p_{\mathrm{CHD}},\;p_{\mathrm{LC}},$ and $p_{\mathrm{BC}},$ respectively. We weighted the objective terms equally to indicate the seriousness of both a recurrence and a fatal late normal tissue complication, but, in a prospective scenario, the priorities of these terms can be adjusted according to the clinician’s or patient’s preference. The models for each penalty function are described below.

While the base analysis in this study focused on O-OPT plans that were optimized purely to minimize $p_{\mathrm{tot}},$ for one patient we also created (i) an O-OPT plan with a hard requirement for ≥90% of CTV receiving the prescription dose and (ii) another O-OPT plan with a hard requirement of avoiding hot spot ≥40 Gy as a sensitivity analysis. Hard requirement (i) was introduced by replacing any dose matrix, achieved by any PSO particle at any optimization iteration that did not satisfy the required tumor coverage by a scaled dose matrix that guaranteed the demanded coverage. In this way, we were enforcing a continuous normalization throughout the iterative optimization process. The details are explained and evaluated in [37]. To enforce hard requirement (ii), any solution not satisfying it during the optimization search process was given an undesirable objective value ($p_{\mathrm{DR}}=$1000%), forcing particles to go away from such solution in the next iteration.

### Disease recurrence model

PFS at 5 years for different dose levels were obtained from randomized trials in literature [1,2,39] assuming uniform irradiation of the CTV. From these studies, hazard ratios (HRs) were estimated at HR = 2.44 for 0 Gy (no RT) relative to 30.6 Gy and HR = 1.44 for 20 Gy relative to 30.6 Gy. Therefore, the estimated PFS at 5 years was given by

(2) $$\mathrm{PFS}(D)=0.872^{\mathrm{HR}(D)}$$

where 0.872 is the observed PFS for full coverage from the 4*x*ABVD (doxorubicin, bleomycin, vinblastine, and dacarbazine) + 30 Gy arm of HD11 trial [2]. HR(D) was calculated by linear interpolation between HR estimates at assumed dose levels of 0, 20, and 30.6 Gy from (1) the HD11 trial reporting HR = 1.49, for 20 Gy versus 30 Gy [27], and (2) Herbst et al.’s [39] review article reporting *HR* = 2.44 for no RT versus CMT. The data from the HD11 trial reflect modern ABVD regimens, while the data comparing chemotherapy alone to CMT include studies of older chemotherapy regimens and may thus slightly overestimate the effect of RT. Also note that the HR extracted from Herbst et al. has been interpreted as representing 0 Gy versus 30.6 Gy, despite several dose regimens in the underlying combined modality arms. The underlying studies mostly used RT doses exceeding 30.6 Gy, except the 20 Gy and 36 Gy arms of the EORTC study where no difference between those dose levels was observed. In the absence of a dose-response above 30.6 Gy we made the pragmatic call to assume HR = 2.44 for 0 versus 30.6 Gy for the present purpose.

The penalty function for risk of disease recurrence was given by

(3) $$p_{\mathrm{DR}}=0.872-\mathrm{PFS}\left(D\right)$$

In the more general case of a non-uniform target dose, the mean dose to the CTV in Equation (2) was calculated as generalized equivalent uniform dose (gEUD) [40]. For voxels with dose >30.6 Gy, dose was capped at 30.6 Gy while for voxels with dose ≤30.6 Gy, dose values were unchanged. This capping of target dose was performed for the calculation of the gEUD so the model did not assume that hot spots in the target were associated with improved local control.

To explore and quantify the common clinical challenge of tradeoffs between target coverage and normal tissue dose, we allowed the O-OPT technique to suggest a target compromise to spare critical risk organs. One approach is to assume that tumor control is a function of mean dose over the target volume. Another common hypothesis is that the minimum dose to the target drives the risk of recurrence. Both situations can be modeled within the formulation of the generalized equivalent uniform dose model suggested by Niemierko, gEUD = ${\left(\frac{1}{n}\sum_{i=1}^{n}{d_{i}}^{a}\right)}^{1/a},$ where *n* is the number of voxels in the CTV, by adjusting the model parameter a [41]. In this formula, setting *a* = 1 corresponds to the mean dose model, whereas a low negative value of a gives high importance of the minimum dose (Figure S2). In this study, we assumed *a* = 1, but also varied the value of a in $\left\{-22,-13,-9,-5,\;-1,\;1\right\}$ as a sensitivity analysis. Figure S2 shows that as a decreases, not irradiating the entire tumor translates to larger $p_{\mathrm{DR}}$ (and therefore larger $p_{\mathrm{tot}}$). This makes it less probable for the optimization algorithm to choose partial irradiation of target for large negative *a*’s.

### Normal tissue complication models

Following Brodin et al. [42], the penalty functions for normal tissue late effect x were assumed to depend on mean dose to the relevant organ (and other patient-specific factors):

(4) $$p_{x}={w_{x}\text{*PFS*}hr}_{\mathrm{excess},x}(D_{x})\int_{e+5yr}^{80\;\mathrm{yr}}{\dot{h}}_{\mathrm{gen}.\mathrm{pop}.,x}\left(a,\mathrm{sex}\right)*\left(S_{\mathrm{gen}}(a,\mathrm{sex})\right)da$$

where $p_{x}$ is the penalty for the optimizer (coronary heart disease [CHD], lung cancer [LC], or breast cancer [BC]). $w_{x}$ is the weighting factor for the mortality associated with x. PFS is calculated in Equation (2). ${hr}_{\mathrm{excess},x}(D_{x})$ is the excess hazard ratio for complication x, which depends on dose $\left(D_{x}\right)$ (Table S1). ${\dot{h}}_{\mathrm{gen}.\mathrm{pop}.,x}\left(a,\mathrm{sex}\right)$ is the population incidence for complication x, which can depend on age (*ɑ*) and sex. $S_{\mathrm{gen}}(a,\mathrm{sex})$ is the sex-specific survival of the general population for each age [43]. The integral is from the age at exposure (e) plus 5 years to 80 years (assuming a latency of late effects of 5 years). The risk of CHD depended on the presence of cardiac risk factors (CRFs) and all patients were optimized twice using different values for ${hr}_{\mathrm{excess},\mathrm{CHD}}$ assuming CRF = 0 and CRF > 0. (For details see Tables S1–S4 [7,8,44–51]).

### Outcome-optimized planning and analysis summary

For each patient, the preplan created in the TPS was exported to the in-house PSO engine where the optimal combination of beams and MUs was determined through minimization of the summed risk of adverse outcomes (modeled as $p_{\mathrm{tot}}$ in Equation (1)). The clinical 3DCRT, VMAT, and O-OPT plans were compared based on their $p_{\mathrm{tot}}$ values as well as dosimetric results; i.e., dose-volume histograms (DVHs) and mean doses to the CTV and the OARs.

## Results

**Figure 2.**
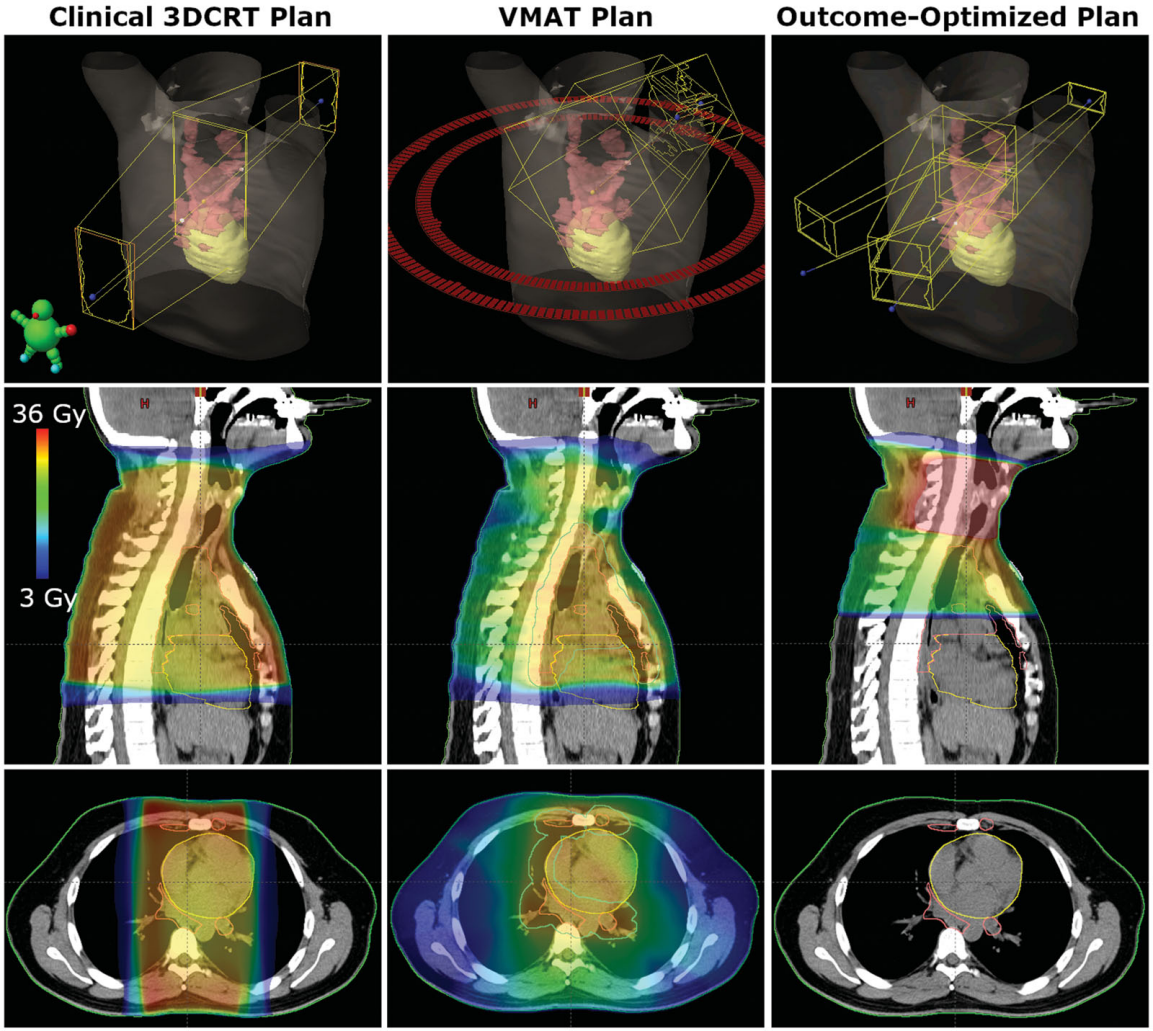
Comparison of beams and dose distributions for clinical 3DCRT, VMAT, and outcome-optimized (O-OPT) plans for an example patient with a large benefit (patient 3 in Table S5). The CTV is shown in pink and the heart is shown in yellow. The PTV is shown in cyan for the VMAT plan.

**Figure 3.**
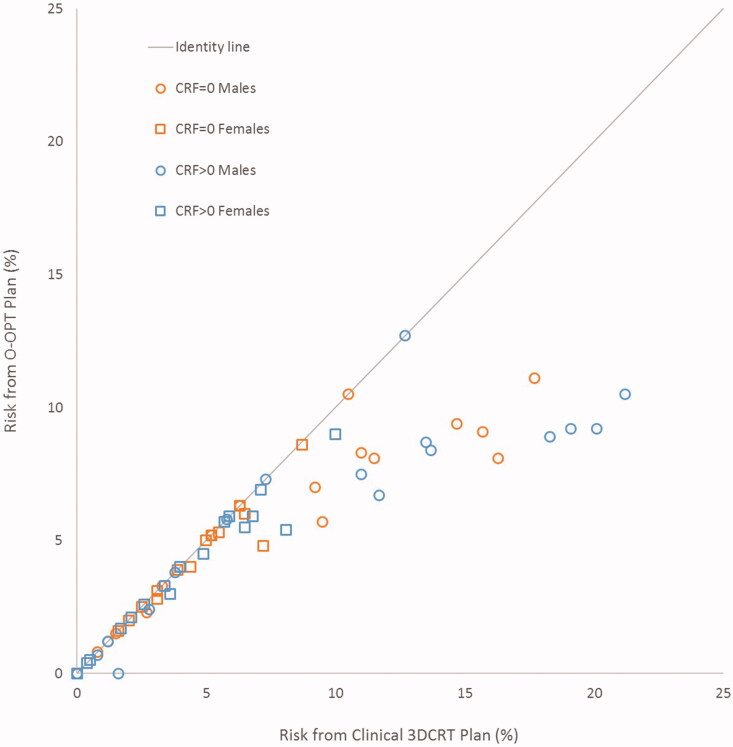
Total risk for outcome-optimized (O-OPT) plans compared to clinical 3DCRT plans for all patients in two cardiac risk factor (CRF) scenarios (CRF = 0 and CRF > 0).

O-OPT plans were created for 34 patients and compared with clinical 3DCRT plans and VMAT plans. In general, O-OPT plans had the lowest risk, followed by the clinical 3DCRT plans, then the VMAT plans with the highest risk with median (maximum) total risk values of 4.9 (11.1), 5.1 (17.7), and 7.6 (20.3)%, respectively (assuming no CRFs) (Tables S5 and S6; Figure S3). Figure S4 gives a graphical representation of sex- and CRF-based data in Tables S5 and S6. Figure 2 shows a comparison between the clinical 3DCRT, VMAT, and O-OPT plans for a patient that had a large modeled benefit from O-OPT planning. O-OPT plans did not provide a risk benefit beyond the clinical 3DCRT plans for 19 of the 34 patients assuming no cardiac risk factors (CRF = 0) (and 13 of 34 assuming CRF > 0), so for those patients the clinical 3DCRT plan was determined to be optimal and chosen as the O-OPT plan. The modeled risk reduction, compared to the clinical 3DCRT plans, was at least 1% for 9 patients assuming no CRFs and for 11 patients assuming the presence of CRFs (Figure S4(a)). Tables S5(a) and S5(b) in the supplementary material summarize the differences between the O-OPT and clinical 3DCRT plans for all patients with and without CRFs, and Table S6 and Figure S4(b) summarize the differences between the O-OPT and VMAT plans. Figure 3 shows the total risk from O-OPT plans compared to clinical 3DCRT plans (Figure S5 shows a comparison with VMAT plans). Median and standard deviation of risk reduction in O-OPT plans versus clinical 3DCRT plans were 0% and 2.3%, respectively, for CRF = 0; and, 0.1% and 3.5%, respectively, for CRF > 0. Median and standard deviation of risk reduction in O-OPT plans versus VMAT plans were 3% and 2.7%, respectively, for CRF = 0.

**Figure 4.**
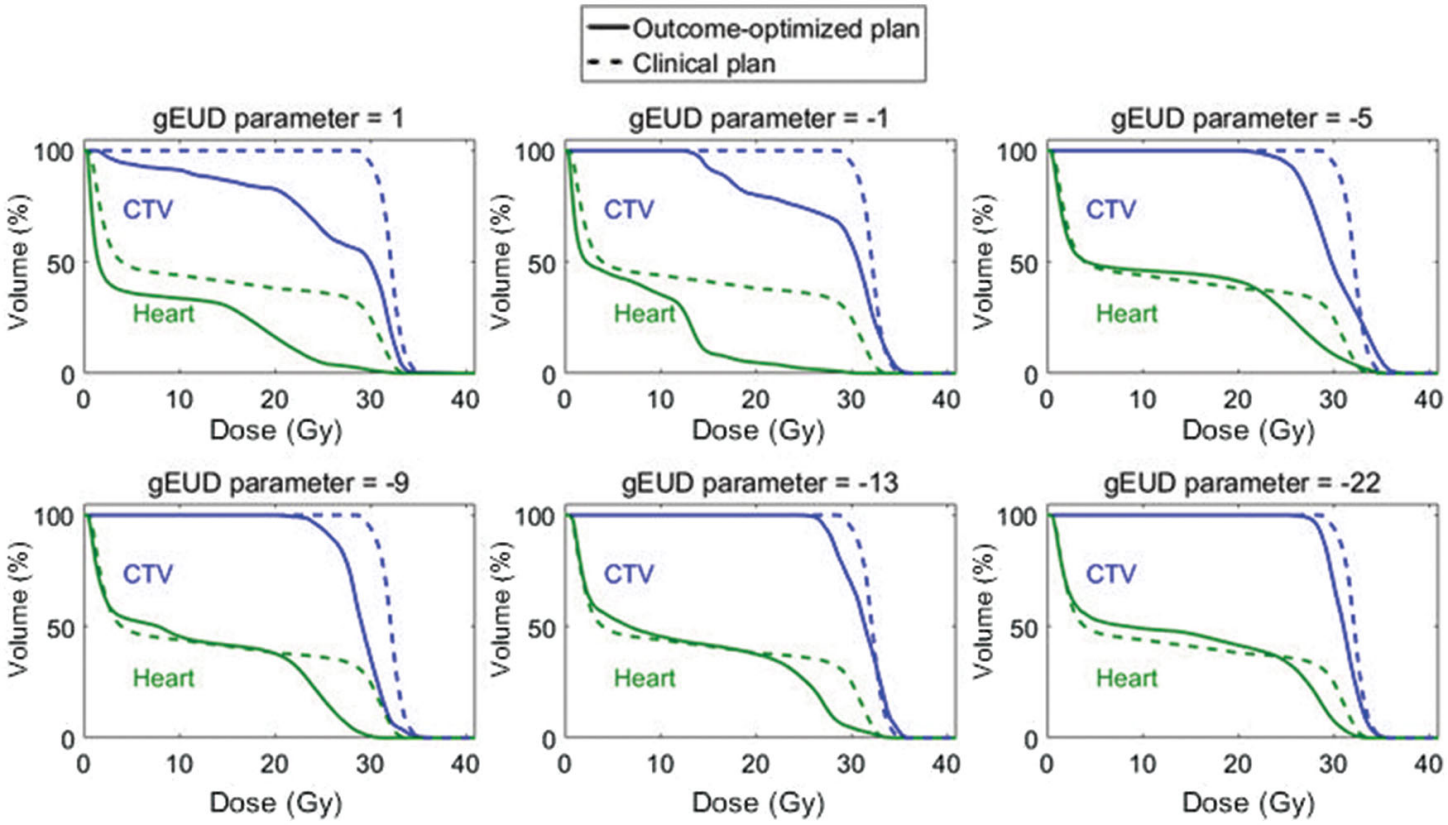
Dose-volume histograms (DVHs) showing the difference in O-OPT plans (CRF = 0 with hot-spot avoidance requirement) with respect to gEUD parameter choice in target model for O-OPT planning for one patient (patient 25). The DVHs from the clinical 3CDRT plan are shown in dotted lines.

The sensitivity analysis for the target gEUD parameter is shown in Figure 4 in an illustrative patient who had a considerable (but not extreme) benefit from O-OPT planning in the base analysis (patient 25). It is seen that by using a larger negative gEUD parameter in the optimization process, the resulting O-OPT plan prioritized target coverage and approached the clinical plan. Figure 4 demonstrates the a-dependent change in the loss of tumor control as tradeoff for reducing fatal normal tissue complications (see also Figure S6 for a visual demonstration of variation in dose distribution). Then, we created O-OPT plans with an extreme value of the gEUD parameter of −22 (which heavily penalized under-dosing any part of the target) for the nine patients who had a benefit from O-OPT planning relative to the clinical 3DCRT plans of at least 1% assuming CRF = 0. Out of these nine patients, only three had a predicted benefit from the O-OPT planning with the gEUD parameter of −22 (Table S7). Furthermore, to investigate the potential increased risk of recurrence if the gEUD parameter was (incorrectly) assumed to be 1 during optimization, we recalculated the risk of recurrence for various (true) values of the gEUD parameter (Table 1) while keeping the plan and dose distribution constant. We found that the unintended increased risk of recurrence due to an incorrect assumption during optimization could be up to 11.7%.

**Table 1.** Recalculation of the term $p_{DR}$ (risk of disease recurrence) for various values of the gEUD parameter *a* for one patient (patient 25).

gEUD parameter a (for recalculation, not optimization)	1	−1	−5	−9	−13	−22
Recalculated $p_{\mathrm{DR}}$ for clinical 3DCRT plan (%)	0.04	0.05	0.05	0.06	0.06	0.07
Recalculated $p_{\mathrm{DR}}$ for O-OPT plan (%)	3.0	8.1	13.9	14.4	14.6	14.8
Increase in $p_{\mathrm{DR}}$ for the O-OPT plan relative to clinical 3DCRT plan for each value of a (O-OPT_a_-3DCRT_a_) (%)	3.0	8.0	13.9	14.4	14.5	14.7
Increase in $p_{\mathrm{DR}}$ for the O-OPT plan for each value of a relative to O-OPT plan with a =1 (O-OPT*_a_*-O-OPT*_a_*_=1_) (%)	0.0	5.0	10.9	11.4	11.6	11.7

For this recalculation, the O-OPT plan that was analyzed was created with the assumption of *a* = 1. Then, the plan was kept constant and $p_{DR}$ was recalculated to see the impact of a different “true” value of *a* if 1 was assumed during optimization but was incorrect.

Finally, we performed a sensitivity analysis with additional requirements during optimization (assuming CRF = 0) for the same patient as the other sensitivity analyses (patient 25). Three O-OPT plans were created for this patient: (1) O-OPT plan (with no extra optimization requirements), (2) O-OPT plan with a target coverage requirement (≥90% of CTV receiving 100% prescribed dose) and (3) O-OPT plan with hot-spot avoidance requirement (40 Gy maximum). Figure S7 compares the DVHs of the clinical 3DCRT plan and the resulting O-OPT plans with the dosimetric requirements. Adding requirements either for target coverage or hot spot avoidance reduced the benefit achievable by O-OPT planning.

## Discussion

In this retrospective study, we investigated the potential of a novel planning approach for patients with mediastinal HL and created individualized plans that aimed to provide the best outcome for the patient by simultaneously optimizing the risk of disease recurrence and mortality due to normal tissue complications. The ultimate aim of this study was not a planning comparison in the traditional sense of the term, but rather to show that including the risk of recurrence and mortality from late toxicity within the optimization function can drastically alter the resulting optimal dose distribution in some patients. While the study contained equal numbers of males and females, 8 of the 9 patients with a benefit ≥1% from O-OPT planning were male. It is possible that the lack of objectives for breast, or the difference in background rates of complications (Tables S2 and S3) influenced this difference. A relationship between benefit from O-OPT planning and age was explored, but no relationship was found. The O-OPT plan was more likely to reduce the risk when the doses to the heart and lungs from the clinical 3DCRT plan or VMAT plan were high (Figure S8). For the patients with the largest doses to the heart and lungs from the clinical 3DCRT plan and largest benefit from O-OPT planning (≥1%), the optimizer would entirely avoid treating the inferior part of the CTV near the heart. Hot spots were observed in O-OPT plans (Figure S9), which were mostly due to the overlap of subfields with collimator rotation from different beam angles or from heavy field weighting from one direction. The total number of beams was comparable in both clinical 3DCRT and O-OPT plans for most patients, so the O-OPT plans would be feasible with respect to delivery time.

These results should be understood within the context of the assumptions and limitations of this study:

First, all risk models reflect our current best evidence regarding dose-risk relationships. Other risk models exist, and more detailed models could become available in the future. Substantial compromises to target coverage were observed in our results. They are, however, driven by our choice of a mean dose model for tumor control probability after an inhomogeneous dose distribution. While we performed a sensitivity analysis of the gEUD model parameter, PFS data after partial target compromise are not available in the literature and this is a major limitation of our approach. A number of alternative modeling approaches for TCP exist including the Poisson model of target cell kill and more elaborate models combining diagnostic accuracy with the original linear-quadratic cell kill formulation [52]. Unfortunately, any such model currently will suffer from the same lack of clinical data to establish model parameters and model uncertainty in the presence of inhomogeneous target doses. Therefore, while this study is a first step in demonstrating the potential of this type of optimization, clinical use should await improved models that account for partial coverage of the CTV. An alternative to including PFS in O-OPT planning would be to constrain the optimizer to provide adequate CTV coverage and only optimize on normal tissue biological endpoints (Figure S7). Further limitations of our PFS model were: (i) chemotherapy regimens were not uniform, and (ii) the definition of disease-free survival might not necessarily be uniform among the studies. However, we did every attempt to focus our data on modern RT, and the 20–30 Gy prescription range is represented by 2 − 4*x*ABVD + RT as delivered in the HD10 and HD11 studies of the German Hodgkin Study Group [1,2].

Second, for the base analysis in this study, no purely dosimetric constraints were used, and we observed dose distributions and maximum doses that were different from usual clinical practice. For example, large maximum doses were seen from subfield overlap and heavy weighting of fields from one direction. When the heavily weighted field was posterior, four plans exceeded the clinical spinal cord constraint of 45 Gy, which may not be acceptable for treatment. Additional optimization objectives might be needed to achieve dose distributions that are also acceptable for the risk of acute toxicity.

Third, the prioritization and weighting were selected to consider disease recurrence to be as equally important as mortality from radiation-induced coronary heart disease, lung cancer, and breast cancer. As the impact of a recurrence on a patient’s quality of life and the high associated risk of mortality might occur earlier than the mortality from a late effect, disease recurrence could theoretically have a time-modulated weighting factor during optimization. Furthermore, non-fatal normal tissue complications could have effects on morbidity and quality of life, which were not modeled here.

Finally, the planning technique limited the degrees of freedom available to create the O-OPT plans. While we provided the optimizer with many fields and allowed simple modulation with subfields, the O-OPT plans were still effectively 3DCRT plans. The limited degrees of freedom (combined with a lack of dosimetric constraints) resulted in hot spots in the O-OPT plans (Figure S9). This O-OPT strategy could be directly integrated with the TPS and combined with VMAT or intensity modulated radiation therapy (IMRT), where it might find more complex solutions with even lower risks.

Despite the above caveats, the approach used in this study, allows a critical examination of the empirical knowledgebase used in treatment plan optimization. It demonstrates the complexities of plan optimization, which is at present carried out in the clinic by crude, semi-quantitative or qualitative methods. Specifically, this individualized dose planning approach represents a framework for considering quantitative estimates of multiple risks, explores the uncertainty in our assumptions, and allows patient-level risk factors to be taken into account. With current capabilities in storing, analyzing, and linking dose plans with treatment outcomes, it is likely that this type of complex, computationally expensive planning will become increasingly reliable in a not too distant future. Importantly, the principles examined in the present study for HL will be applicable to other tumor types. However, the importance of local tumor control relative to the importance of different toxicities must be viewed in the context of each individual tumor type as well as each individual patient. In tumor types, e.g., head and neck cancer, where the relevant morbidities, although causing significant symptoms, are rarely lethal, and where the risk of local recurrence is higher and no curative salvage treatment is available, the weights assigned to the different outcomes will have to be modified accordingly.

In conclusion, we investigated an RT planning strategy where we directly optimized on a metric for patient-specific outcome. Total risk was defined as an equally weighted summation of risks of disease recurrence and mortality due to radiation-induced coronary heart disease and secondary lung and breast cancers. Our technique reduced the maximum total risk considerably for patients who had large OAR doses in their VMAT plans or clinical 3DCRT plans; however, for patients with relatively low OAR doses in clinical 3DCRT plans, there was no improvement achieved through O-OPT planning. Sensitivity analyses investigating dependence of our results on the TCP model (the gEUD parameter) and dosimetric requirements revealed large variation in both plan result and risk of recurrence, demonstrating a need for caution in future work on biologically optimized planning for HL.

## Supplementary Material

Supplemental Material
