# Supplementary material for: Biological optimization for mediastinal lymphoma radiotherapy – a preliminary study
Source: Acta Oncol. 2020 Mar 27;59(8):879–87. doi: 10.1080/0284186X.2020.1733654 (PMC7446040; doi:10.1080/0284186X.2020.1733654)
Supplement: Supplemental Material [file IONC_A_1733654_SM1953.pdf]

Biological optimization for mediastinal lymphoma radiotherapy – a preliminary study

Supplementary material

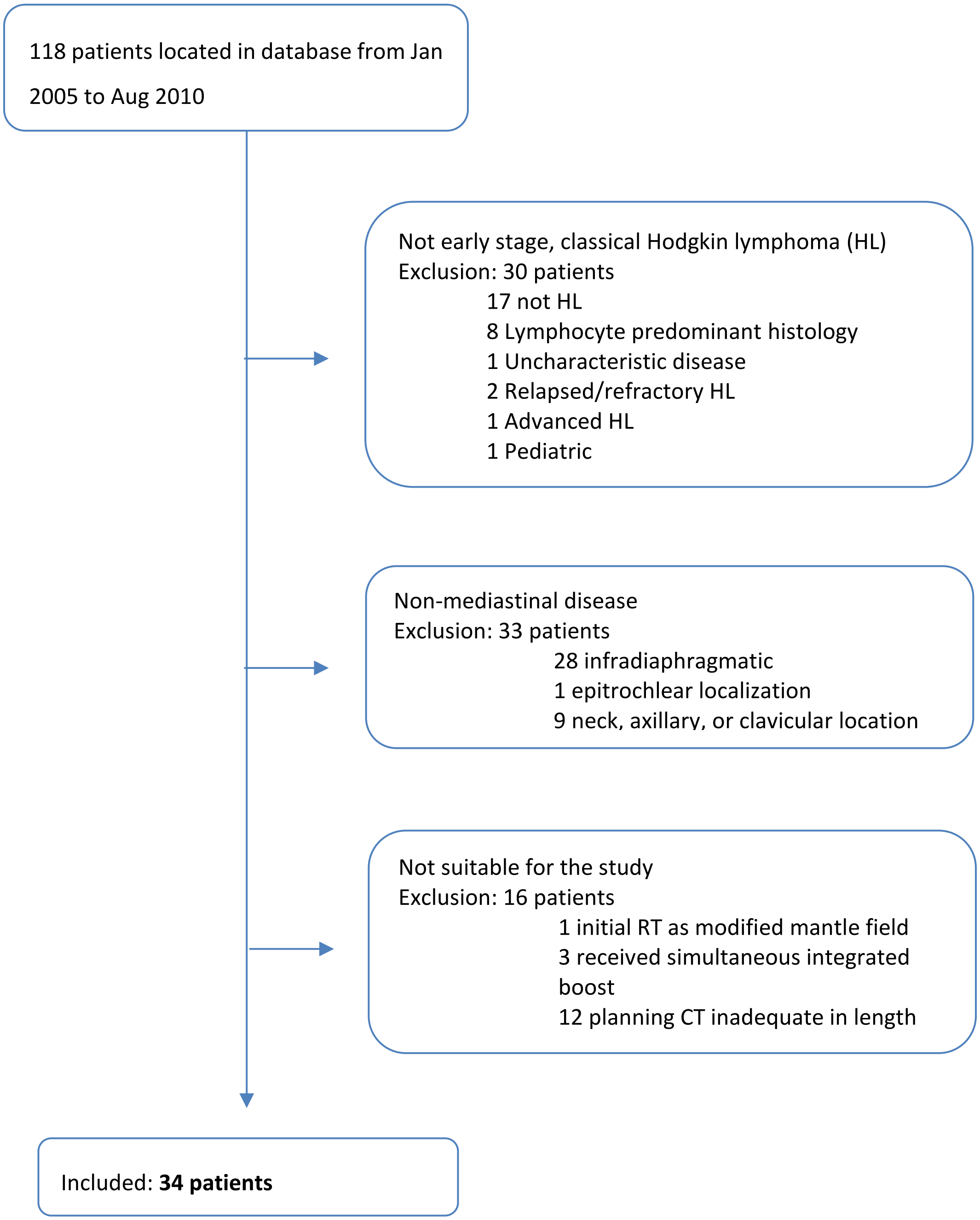

Figure S1: Flowchart of patient inclusion in this study [7].

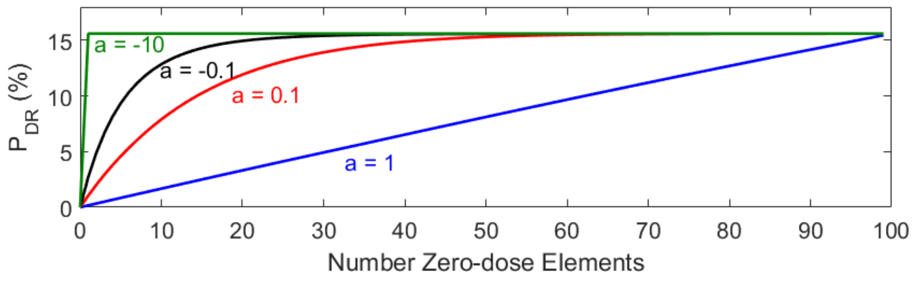

Figure S2. Illustration of the impact of using gEUD [18,19] with various parameter (a) values in recurrence risk calculation (P_{DR} is the penalty for disease risk). A 100-voxel tumor model is considered for this example.

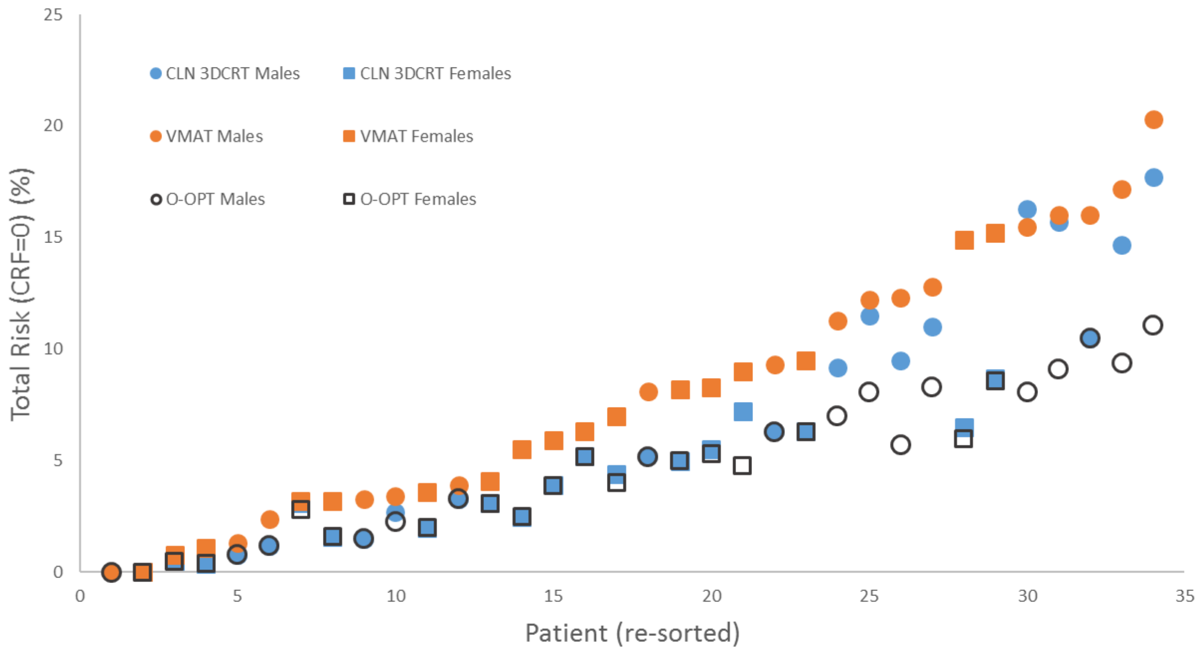

Figure S3. Total risk (recurrence and mortality from late effects) for each patient for each type of plan: clinical (CLN) 3DCRT, VMAT, and O-OPT (assuming CRF=0). Patients are sorted by VMAT risk. Male patients are shown as circles and female patients are shown as squares.

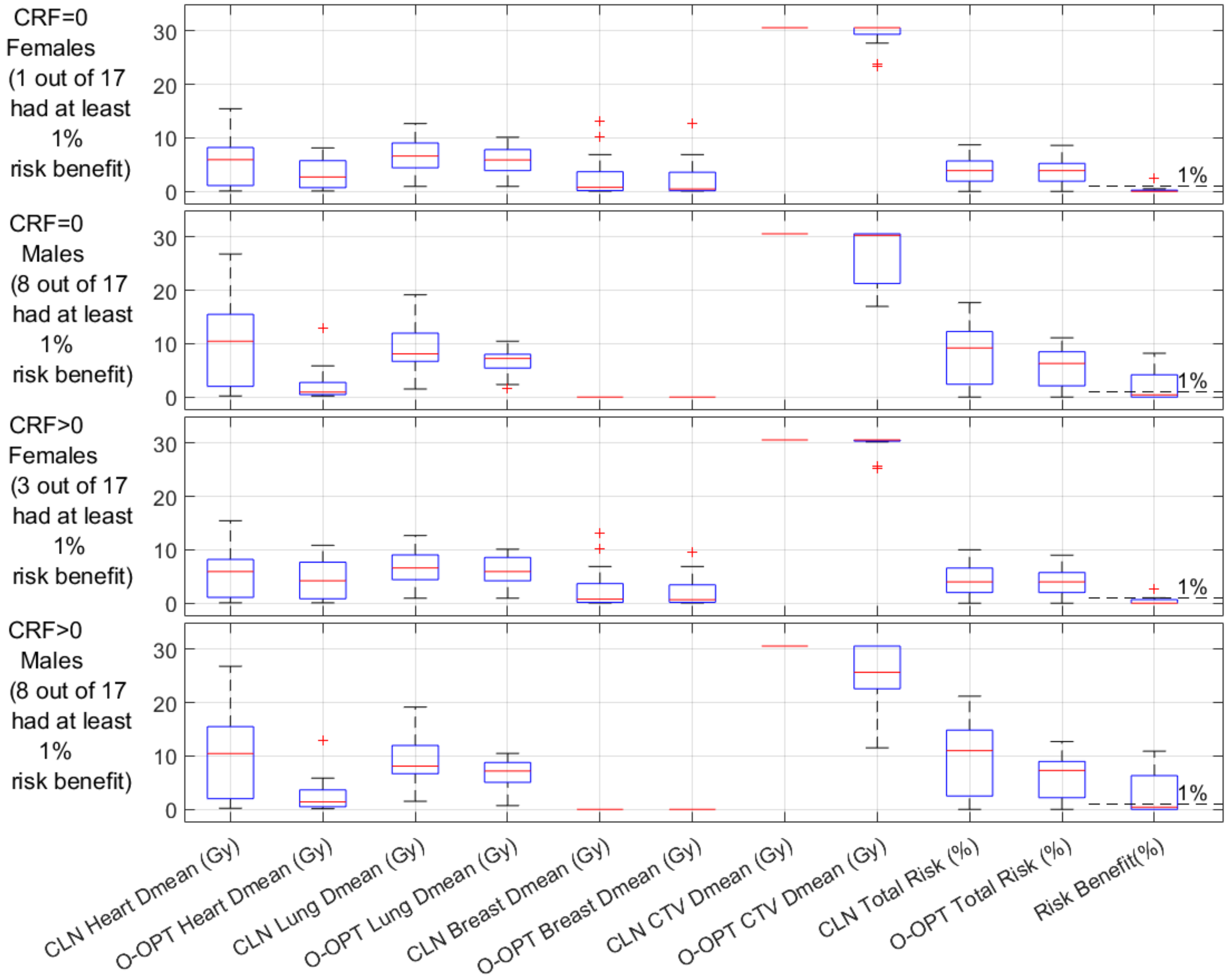

Figure S4(a.1). A sex-based and CRF-based representation of the results in Tables S5(a) and S5(b).

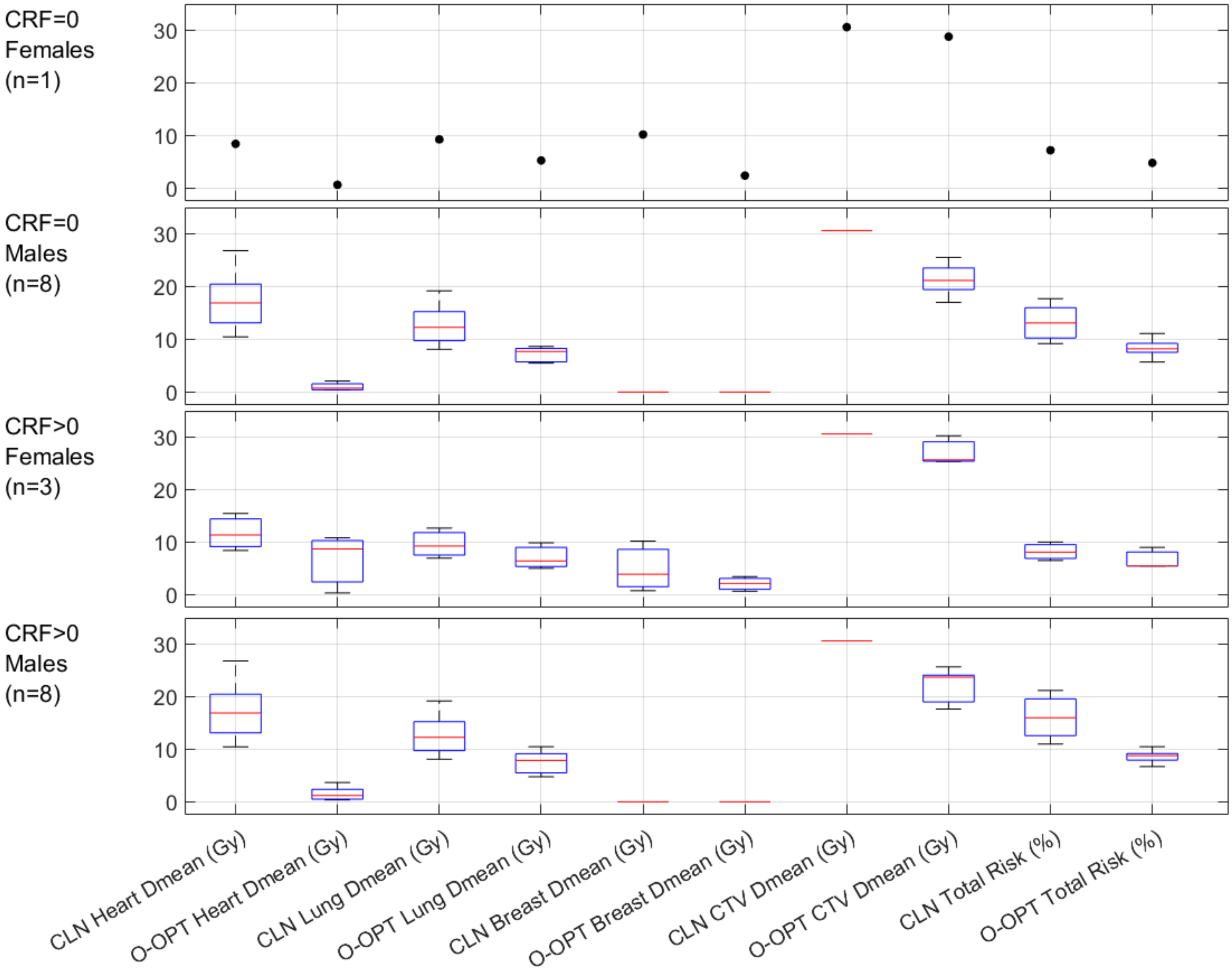

Figure S4(a.2). A sex-based and CRF-based representation of the results in Tables S5(a) and S5(b) only for patient cases where at least 1% risk benefit was achieved.

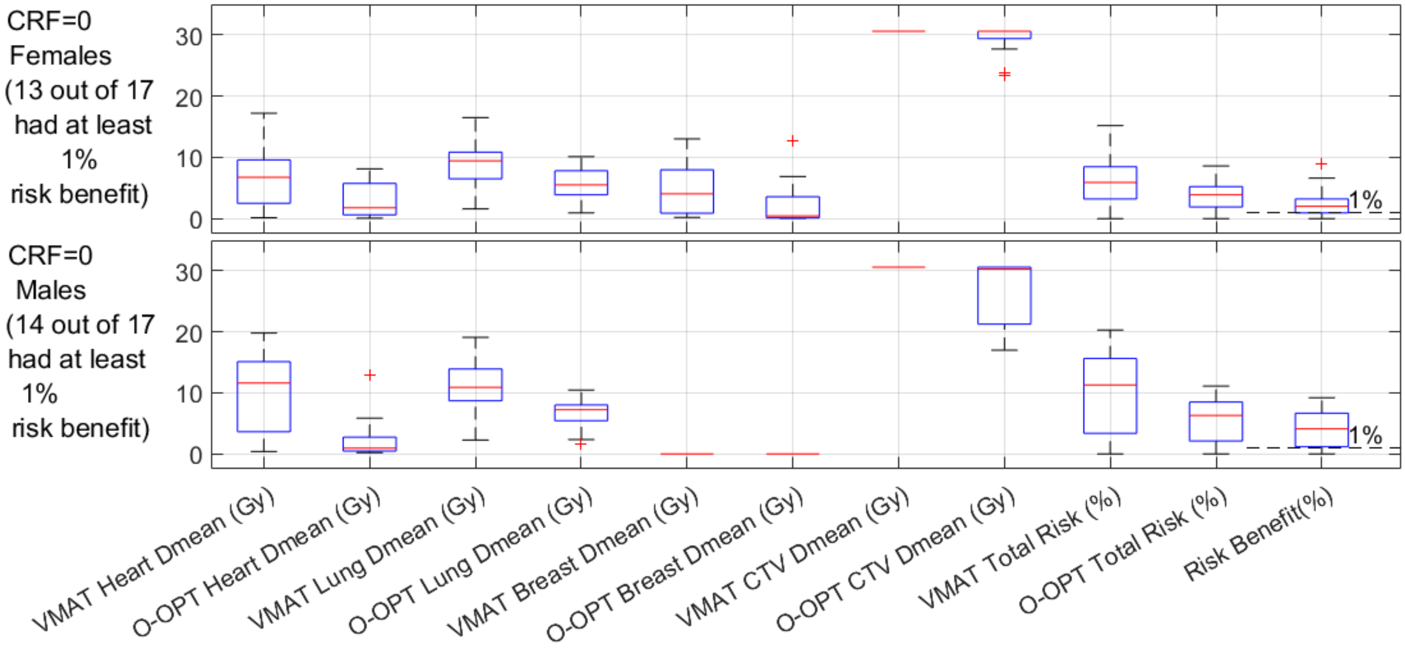

Figure S4(b.1). A sex-based and CRF-based representation of the results in Table S6.

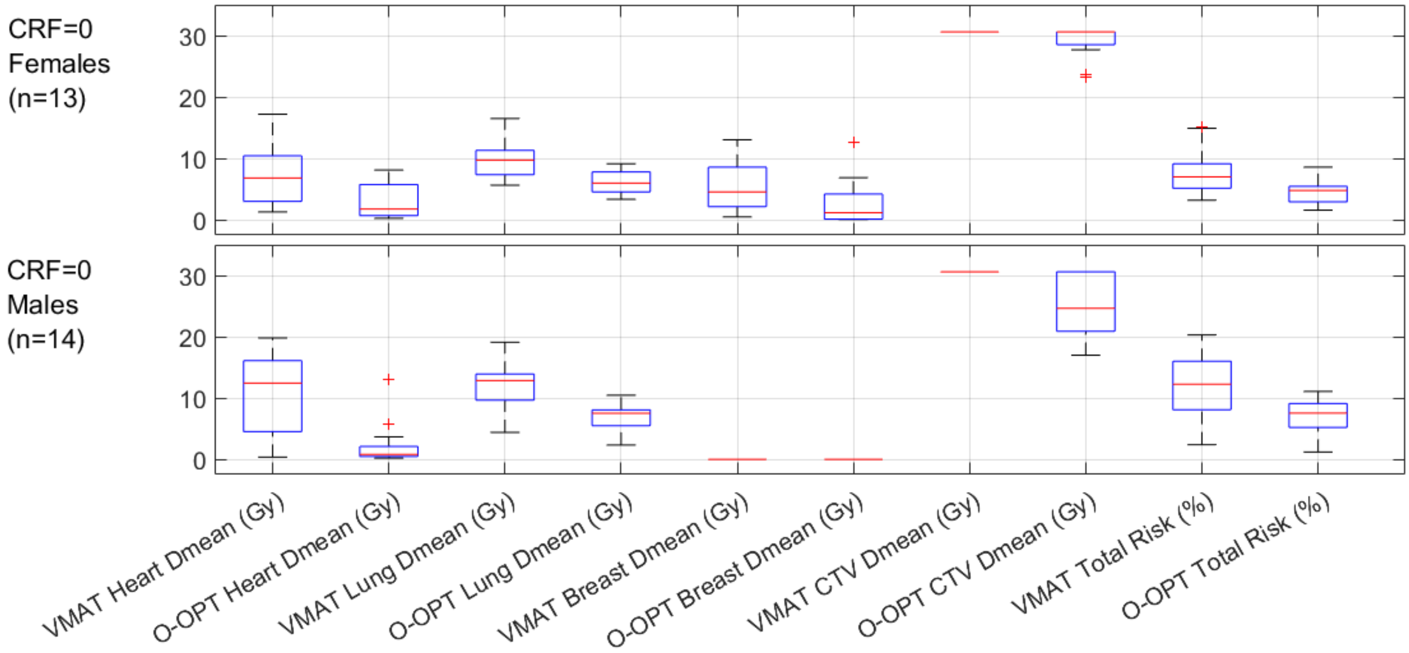

Figure S4(b.2). A sex-based and CRF-based representation of the results in Table S6 only for patient cases where at least 1% risk benefit was achieved.

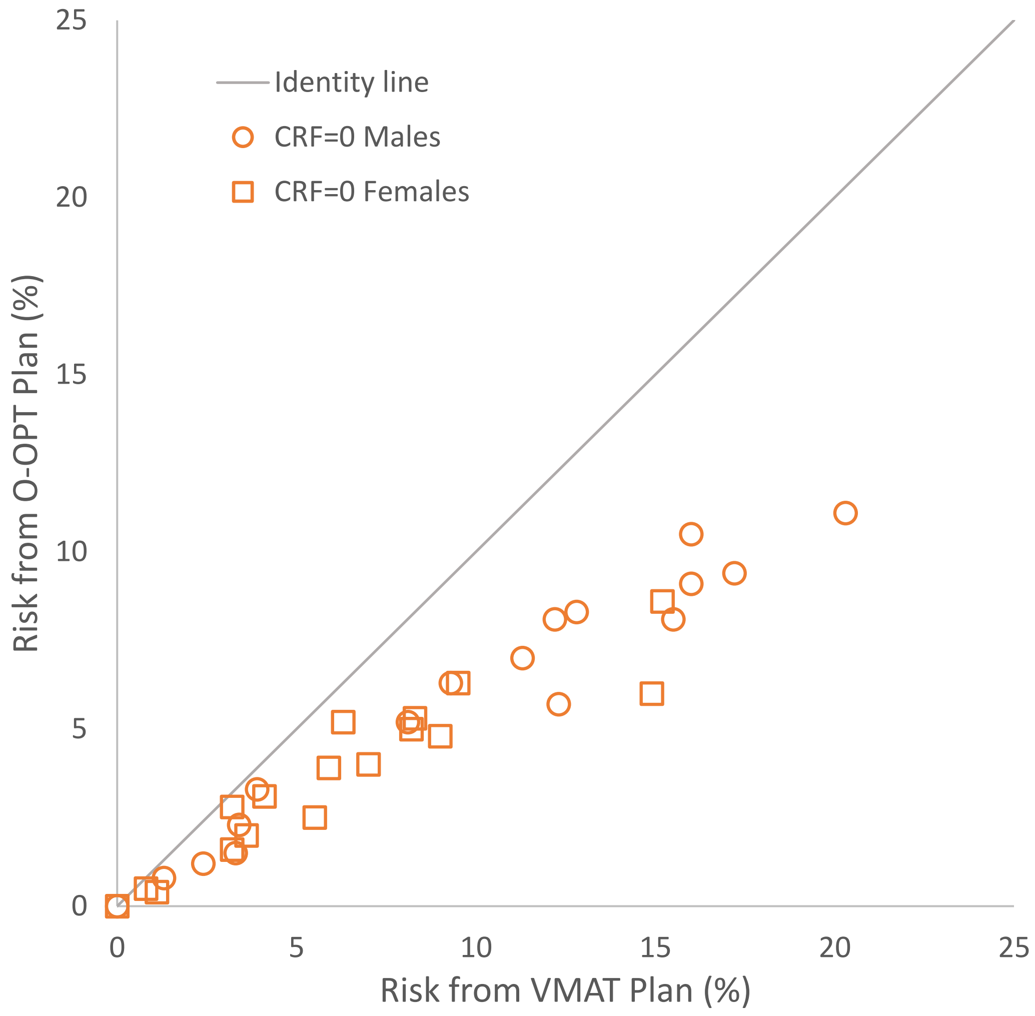

Figure S5. Total risk for outcome-optimized (O-OPT) plans compared to VMAT plans for all patients (CRF=0). Male patients are shown as circles and female patients are shown as squares.

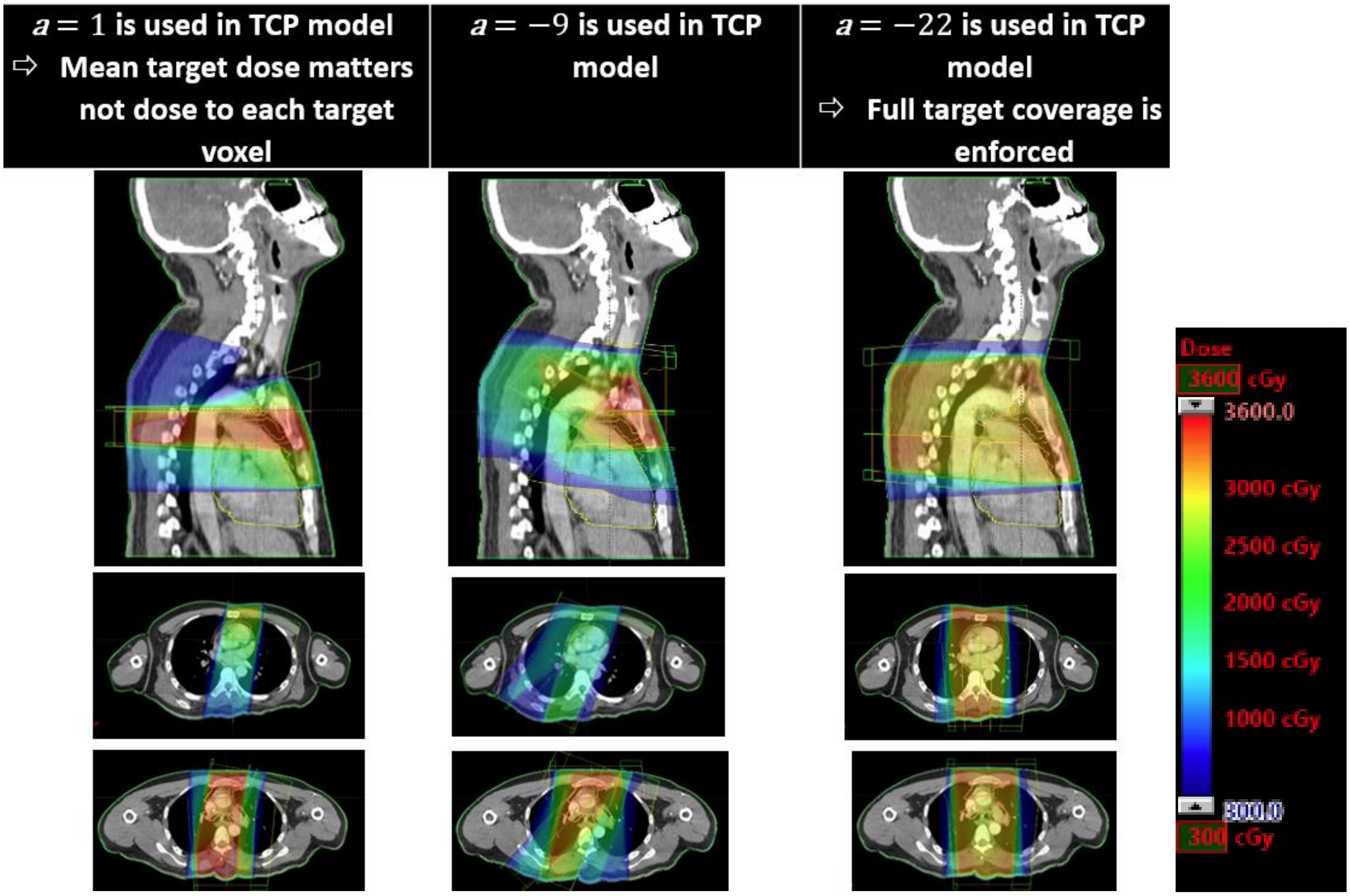

Figure S6. An example of variation in dose distribution when different gEUD parameter values are used in the TCP model in the plan optimization process.

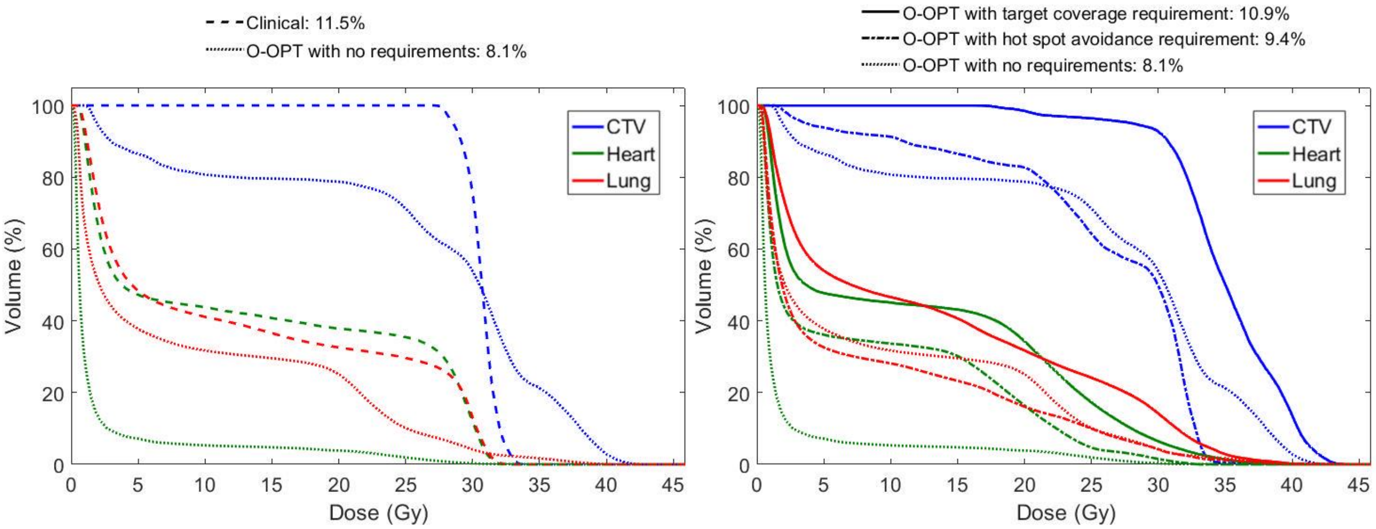

Figure S7. Change of dose-volume histogram (DVH) with respect to target coverage and hot-spot avoidance requirements in outcome-optimized (O-OPT) planning (CFR=0) for one patient. The total risk for each is listed in the legend. Clinical plan for comparison is the 3DCRT plan.

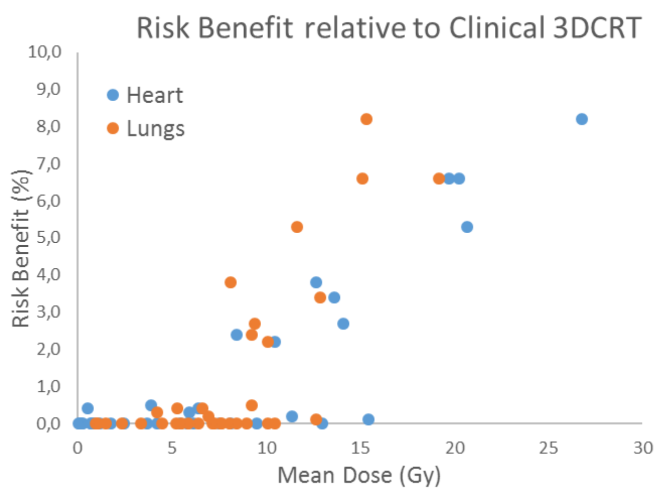

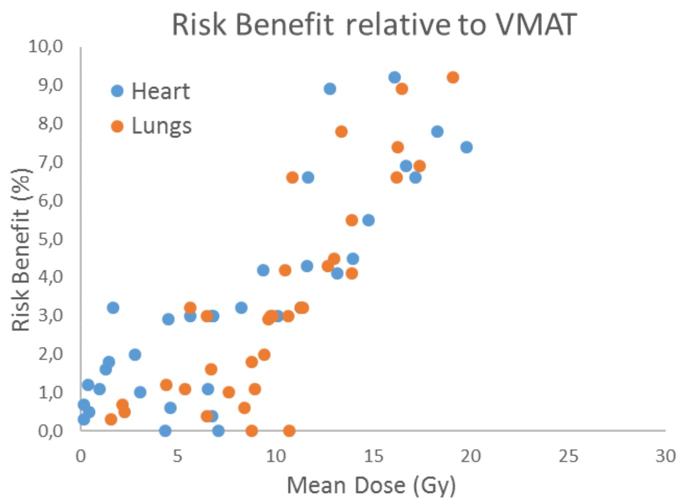

Figure S8. The total risk benefit from O-OPT planning compared to the mean dose to the heart or lungs from the clinical 3DCRT plan (left) or VMAT plan (right), (CRF=0).

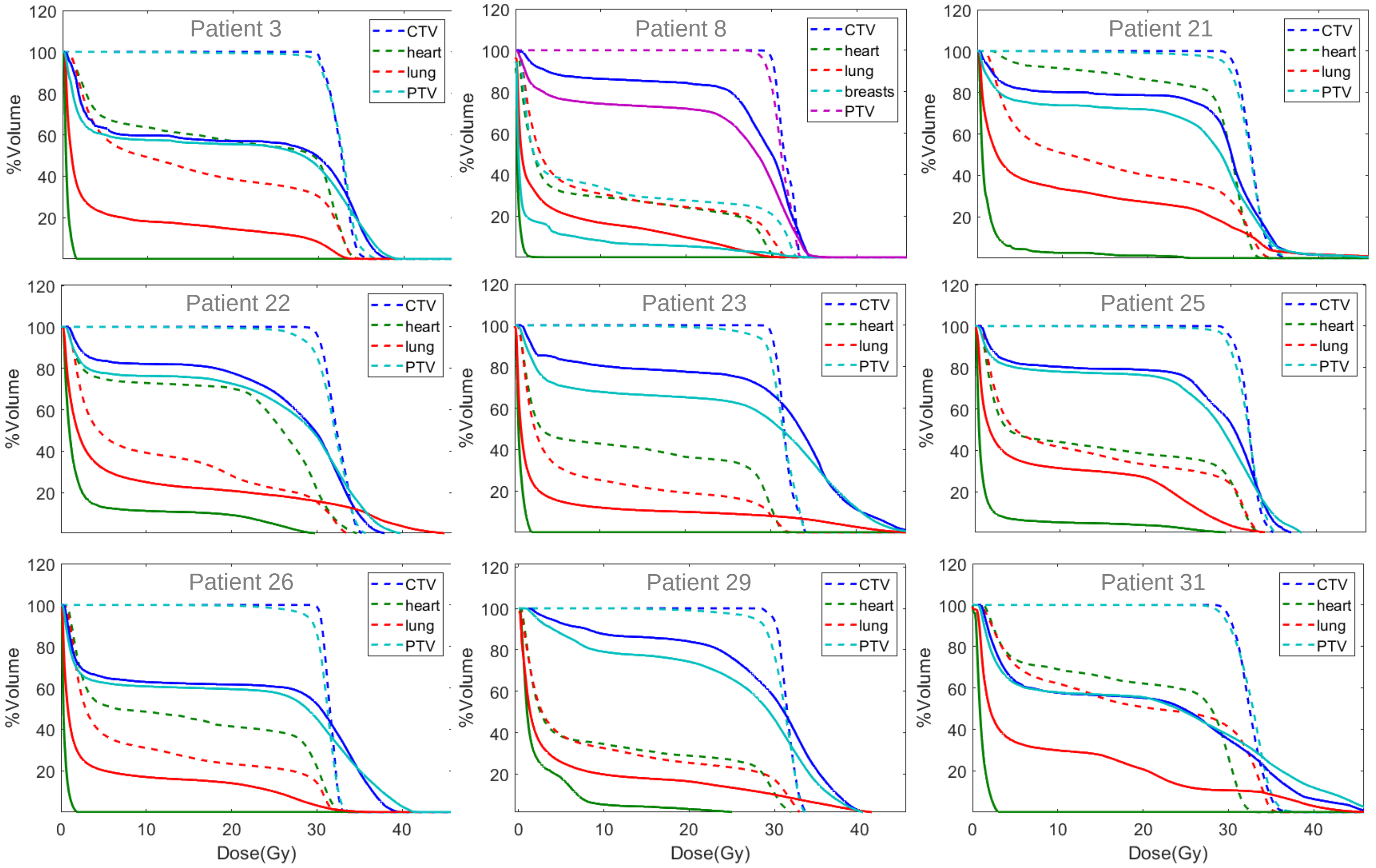

Figure S9. Dose-volume histograms for the clinical 3DCRT plan (dashed) compared to the O-OPT plan (solid) for CRF=0 for the 9 patients with benefit >1%.

Table S1. The risk models used to calculate the excess hazard ratios (hr_{excess}) of the normal tissue complications in this study. Abbreviations: MHD: mean heart dose (Gy), MLD: mean lung dose (Gy), MBD: mean breast dose (Gy) of glandular tissue, CRF. Cardiac risk factor.

**Complication (x)**	$hr_{excess,x}(male)$	$hr_{excess,x}(female)$	**Reference**
Coronary Heart Disease (CHD)	0.070*MHD (CRF=0)<br>0.097*MHD (CRF>0)	0.070*MHD (CRF=0)<br>0.097*MHD (CRF>0)	van Nimwegen et al 2016 [36]
Lung Cancer (LC)	0.141*MLD	0.141*MLD	Travis et al 2002[34]
Breast Cancer (BC)	N/A	0.149*MBD	Travis et al 2003[35]

Table S2: Background death rates from all heart disease (HD) [37]

Age range (a) (years)	$\dot{h}_{gen.pop.,HD}(a, male)$ (rate per year %)	$\dot{h}_{gen.pop.,HD}(a, female)$ (rate per year %)
15-24	0.0027	0.0016
25-34	0.0103	0.0050
35-44	0.0348	0.0164
45-54	0.1135	0.0475
55-64	0.2681	0.1093
65-74	0.5257	0.2617
75-84	1.3548	0.8548

The ratio between the death rates of coronary heart disease (CHD) and death rates of all heart disease (HD) was calculated by assuming that this ratio is not age dependent [38]:

$$w_{CHD}(male) = \frac{death\ rate\ of\ CHD}{death\ rate\ of\ HD} = \frac{133.5}{210.9} = 0.63$$

$$w_{CHD}(female) = \frac{death\ rate\ of\ CHD}{death\ rate\ of\ HD} = \frac{71.6}{131.8} = 0.54$$

Table S3: Background rates of lung cancer [39,40]

Age range (a) (years)	$\dot{h}_{gen.pop.,LC}(a, male)$ (% per year)	$\dot{h}_{gen.pop.,LC}(a, female)$ (% per year)
20-29	0.000	0.001
30-39	0.002	0.002
40-49	0.014	0.014
50-59	0.067	0.056
60-69	0.190	0.146
70-79	0.344	0.263

The weighting factor for mortality for lung cancer was 1 minus the 5 year survival of lung cancer [41]:

w_{LC} = 1-0.177 = 0.823.

Table S4. Background rates of breast cancer [42]

Age range (a) (years)	$\dot{h}_{gen.pop.,BC}(a, female)$ (% per year)
20-29	0.006
30-39	0.045
40-49	0.147
50-59	0.230
60-69	0.347
70-79	0.395

The weighting factor for mortality for breast cancer was 1 minus the 5-year survival rate of breast cancer [43]:

w_{BC} = 1-0.897 = 0.103.

Table S5(a). Dosimetric and risk comparison between the clinical (CLN) 3DCRT plans and outcome-optimized (O-OPT) plans for no cardiac risk factors (CRF=0).

Patient Info				Heart D_{mean} (Gy)		Lung D_{mean} (Gy)		Breast D_{mean} (Gy)		CTV D_{mean} (Gy)		Total Risk (%)		Risk Benefit (%)	CLN 3DCRT plan chosen as O-OPT
ID	sex	age	CTV (cc)	CLN 3DCRT	O-OPT	CLN 3DCRT	O-OPT	CLN 3DCRT	O-OPT	CLN 3DCRT	O-OPT	CLN 3DCRT	O-OPT	3DCRT – O-OPT	
1	F	24	106.6	6.41	5.80	6.60	5.98	1.74	1.17	30.60	30.24	4.4	4.0	0.4	
2	M	22	298.9	3.66	3.66	7.66	7.66	0.00	0.00	30.60	30.60	5.2	5.2	0	X
3	M	32	561.4	19.73	0.54	15.12	5.79	0.00	0.00	30.60	18.51	15.7	9.1	6.6	
4	F	22	44.2	0.06	0.06	0.93	0.93	0.04	0.04	30.60	30.60	0.4	0.4	0	X
5	M	46	70.5	0.26	0.26	1.51	1.51	0.00	0.00	30.60	30.60	0.8	0.8	0	X
6	M	41	61.3	0.21	0.21	2.34	2.34	0.00	0.00	30.60	30.60	1.2	1.2	0	X
7	M	76	122.8	2.43	2.43	7.18	7.18	0.00	0.00	30.60	30.60	0.0	0.0	0	X
8	F	22	161.2	8.42	0.65	9.27	5.26	10.2	2.39	30.60	28.79	7.2	4.8	2.4	
9	F	36	39.5	5.92	4.16	4.20	3.66	0.58	0.42	30.60	29.58	3.1	2.8	0.3	
10	F	25	130.8	1.16	1.16	5.51	5.51	0.15	0.15	30.60	30.60	2.5	2.5	0	X
11	M	63	174	5.44	5.44	7.24	7.24	0.00	0.00	30.60	30.60	3.3	3.3	0	X
12	F	43	644.7	15.46	3.41	12.67	9.13	3.89	5.90	30.60	23.72	8.7	8.6	0.1	
13	F	27	197	0.85	0.85	4.48	4.48	0.13	0.13	30.60	30.60	2.0	2.0	0	X
14	F	35	123.5	1.77	1.77	6.39	6.39	6.86	6.86	30.60	30.60	3.9	3.9	0	X
15	F	18	1097	3.87	2.68	9.23	8.13	13.20	12.63	30.60	29.83	6.5	6.0	0.5	
16	F	27	160.9	9.50	5.73	8.98	7.71	3.60	3.67	30.60	27.72	6.3	6.3	0	X
17	M	22	85.8	0.50	0.48	5.27	4.19	0.00	0.00	30.60	30.31	2.7	2.3	0.4	
18	M	31	213.6	5.84	5.84	8.08	8.08	0.00	0.00	30.60	30.60	6.3	6.3	0	X
19	F	30	28.6	0.10	0.10	1.08	1.08	0.08	0.08	30.60	30.60	0.5	0.5	0	X
20	F	43	197.5	7.52	7.52	7.61	7.61	3.54	3.54	30.60	30.60	5.0	5.0	0	X
21	M	52	777.6	26.81	1.25	15.37	8.55	0.00	0.00	30.60	20.38	16.3	8.1	8.2	
22	M	16	584.4	20.67	0.94	11.66	8.00	0.00	0.00	30.60	21.42	14.7	9.4	5.3	
23	M	17	277.1	12.65	0.46	8.10	5.67	0.00	0.00	30.60	23.24	9.5	5.7	3.8	
24	M	36	325.2	13.01	13.01	10.46	10.46	0.00	0.00	30.60	30.6	10.5	10.5	0	X
25	M	44	227.1	13.61	1.88	12.87	8.66	0.00	0.00	30.60	23.8	11.5	8.1	3.4	
26	M	22	415.7	14.1	0.42	9.39	5.5	0.00	0.00	30.60	20.93	11.0	8.3	2.7	
27	F	40	117	8.13	8.13	8.45	8.45	0.31	0.31	30.60	30.60	5.2	5.2	0	X
28	F	51	96.1	4.20	4.20	5.89	5.89	1.27	1.27	30.60	30.60	3.1	3.1	0	X
29	M	38	330.4	10.46	2.12	10.12	8.02	0.00	0.00	30.60	25.52	9.2	7.0	2.2	
30	F	17	138.2	11.36	0.26	6.95	3.96	0.75	0.08	30.60	23.30	5.5	5.3	0.2	
31	M	35	1559	20.27	0.41	19.18	7.38	0.00	0.00	30.60	17.00	17.7	11.1	6.6	
32	F	76	136.4	6.16	6.16	10.11	10.11	0.44	0.44	30.60	30.60	0.0	0.0	0	X
33	M	64	219.1	0.69	0.69	5.25	5.25	0.00	0.00	30.60	30.60	1.5	1.5	0	X
34	F	18	113.1	0.73	0.73	3.37	3.37	0.13	0.13	30.60	30.60	1.6	1.6	0	X
**median**		**33.5**	**167.6**	**6.04**	**1.51**	**7.64**	**6.19**	**0.02**	**0.02**	**30.6**	**30.6**	**5.1**	**4.9**	**0**	
**Stdev***		**16.3**	**324.6**	**7.05**	**2.99**	**4.13**	**2.48**	**3.05**	**2.65**	**0.0**	**4.2**	**5.0**	**3.2**	**2.3**	

* Stdev: Standard deviation

Table S5(b). Dosimetric and risk comparison between the clinical (CLN) 3DCRT plans and outcome-optimized (O-OPT) plans for cardiac risk factors present (CRF>0).

Patient Info				Heart D_{mean} (Gy)		Lung D_{mean} (Gy)		Breast D_{mean} (Gy)		CTV D_{mean} (Gy)		Total Risk (%)		Risk Benefit (%)	CLN 3DCRT plan chosen as O-OPT
ID	sex	age	CTV (cc)	CLN 3DCRT	O-OPT	CLN 3DCRT	O-OPT	CLN 3DCRT	O-OPT	CLN 3DCRT	O-OPT	CLN 3DCRT	O-OPT	3DCRT – O-OPT	
1	F	24	106.6	6.41	6.05	6.60	5.94	1.74	0.71	30.60	30.32	4.9	4.5	0.4	
2	M	22	298.9	3.66	3.66	7.66	7.66	0.00	0.00	30.60	30.60	5.8	5.8	0	X
3	M	32	561.4	19.73	0.46	15.12	5.89	0.00	0.00	30.60	18.59	19.1	9.2	9.9	
4	F	22	44.2	0.06	0.06	0.93	0.93	0.04	0.04	30.60	30.60	0.4	0.4	0	X
5	M	46	70.5	0.26	0.11	1.51	0.70	0.00	0.00	30.60	11.54	0.8	0.7	0.1	
6	M	41	61.3	0.21	0.21	2.34	2.34	0.00	0.00	30.60	30.60	1.2	1.2	0	X
7	M	76	122.8	2.43	2.43	7.18	7.18	0.00	0.00	30.60	30.60	0	0	0	X
8	F	22	161.2	8.42	0.33	9.27	5.02	10.2	2.13	30.60	25.34	8.1	5.4	2.7	
9	F	36	39.5	5.92	5.71	4.20	3.42	0.58	0.58	30.60	30.52	3.6	3.0	0.6	
10	F	25	130.8	1.16	1.16	5.51	5.51	0.15	0.15	30.60	30.60	2.6	2.6	0	X
11	M	63	174	5.44	5.44	7.24	7.24	0.00	0.00	30.60	30.60	3.8	3.8	0	X
12	F	43	644.7	15.46	8.71	12.67	9.88	3.89	3.43	30.60	25.68	10.0	9.0	1	
13	F	27	197	0.85	0.85	4.48	4.48	0.13	0.13	30.60	30.60	2.1	2.1	0	X
14	F	35	123.5	1.77	1.77	6.39	6.39	6.86	6.86	30.60	30.60	4.0	4.0	0	X
15	F	18	1097	3.87	2.58	9.23	9.07	13.20	9.53	30.60	30.40	6.8	5.9	0.9	
16	F	27	160.9	9.50	9.50	8.98	8.98	3.60	3.60	30.60	30.60	7.1	6.9	0.2	
17	M	22	85.8	0.50	0.49	5.27	4.26	0.00	0.00	30.60	30.38	2.8	2.4	0.4	
18	M	31	213.6	5.84	5.84	8.08	8.08	0.00	0.00	30.60	30.60	7.3	7.3	0	X
19	F	30	28.6	0.10	0.10	1.08	1.08	0.08	0.08	30.60	30.60	0.5	0.5	0	X
20	F	43	197.5	7.52	7.52	7.61	7.61	3.54	3.54	30.60	30.60	5.7	5.7	0	X
21	M	52	777.6	26.81	1.41	15.37	10.48	0.00	0.00	30.60	23.76	20.1	9.2	10.9	
22	M	16	584.4	20.67	3.69	11.66	9.19	0.00	0.00	30.60	23.68	18.3	8.9	9.4	
23	M	17	277.1	12.65	0.55	8.10	4.78	0.00	0.00	30.60	24.38	11.7	6.7	5	
24	M	36	325.2	13.01	13.01	10.46	10.46	0.00	0.00	30.60	30.6	12.7	12.7	0	X
25	M	44	227.1	13.61	1.89	12.87	8.68	0.00	0.00	30.60	23.82	13.7	8.4	5.3	
26	M	22	415.7	14.1	0.37	9.39	5.16	0.00	0.00	30.60	19.40	13.5	8.7	4.8	
27	F	40	117	8.13	8.13	8.45	8.45	0.31	0.31	30.60	30.60	5.9	5.9	0	X
28	F	51	96.1	4.20	4.20	5.89	5.89	1.27	1.27	30.60	30.60	3.4	3.3	0.1	
29	M	38	330.4	10.46	2.86	10.12	7.08	0.00	0.00	30.60	25.69	11.0	7.5	3.5	
30	F	17	138.2	11.36	10.85	6.95	6.41	0.75	0.64	30.60	30.25	6.5	5.5	1	
31	M	35	1559	20.27	0.98	19.18	9.14	0.00	0.00	30.60	17.65	21.2	10.5	10.7	
32	F	76	136.4	6.16	6.16	10.11	10.11	0.44	0.44	30.60	30.60	0.0	0.0	0	X
33	M	64	219.1	0.69	0.69	5.25	5.25	0.00	0.00	30.60	30.60	1.6	1.6	0	X
34	F	18	113.1	0.73	0.73	3.37	3.37	0.13	0.13	30.60	30.60	1.7	1.7	0	X
**median**		**33.5**	**167.6**	**6.04**	**2.51**	**7.64**	**6.40**	**0.02**	**0.06**	**30.6**	**30.6**	**5.8**	**5.5**	**0.1**	
**stdev**		**16.3**	**324.6**	**7.05**	**3.51**	**4.13**	**2.94**	**3.05**	**5.50**	**0.0**	**6.6**	**6.1**	**3.5**	**3.5**	

Table S6. Dosimetric and risk comparison between the VMAT plans and outcome-optimized (O-OPT) plans for no cardiac risk factors (CRF = 0).

Patient Info				Heart D_{mean} (Gy)		Lung D_{mean} (Gy)		Breast D_{mean} (Gy)		CTV D_{mean} (Gy)		Total Risk (%)		Risk Benefit (%)	CLN 3DCRT plan chosen as O-OPT
ID	sex	age	CTV (cc)	VMAT	O-OPT	VMAT	O-OPT	VMAT	O-OPT	VMAT	O-OPT	VMAT	O-OPT		
1	F	24	106.6	6.82	5.80	9.84	5.98	4.56	1.17	30.6	30.2	7.0	4.0	3.0	
2	M	22	298.9	4.53	3.66	9.65	7.66	0.00	0.00	30.6	30.6	8.1	5.2	2.9	X
3	M	32	561.4	16.7	0.54	17.4	5.79	0.00	0.00	30.6	18.5	16.0	9.1	6.9	
4	F	22	44.2	0.16	0.06	2.17	0.93	0.20	0.04	30.6	30.6	1.1	0.4	0.7	X
5	M	46	70.5	0.43	0.26	2.28	1.51	0.00	0.00	30.6	30.6	1.3	0.8	0.5	X
6	M	41	61.3	0.38	0.21	4.41	2.34	0.00	0.00	30.6	30.6	2.4	1.2	1.2	X
7	M	76	122.8	4.36	2.43	8.81	7.18	0.00	0.00	30.6	30.6	0.0	0.0	0.0	X
8	F	22	161.2	9.4	0.65	10.5	5.26	8.56	2.39	30.6	28.8	9.0	4.8	4.2	
9	F	36	39.5	6.75	4.16	6.50	3.66	4.04	0.42	30.6	29.6	3.2	2.8	0.4	
10	F	25	130.8	6.74	1.16	6.49	5.51	4.04	0.15	30.6	30.6	5.5	2.5	3.0	X
11	M	63	174	4.60	5.44	8.42	7.24	0.00	0.00	30.6	30.6	3.9	3.3	0.6	X
12	F	43	644.7	17.2	3.41	16.2	9.13	11.0	5.90	30.6	23.7	15.2	8.6	6.6	
13	F	27	197	1.30	0.85	6.72	4.48	0.89	0.13	30.6	30.6	3.6	2.0	1.6	X
14	F	35	123.5	2.79	1.77	9.43	6.39	7.77	6.86	30.6	30.6	5.9	3.9	2.0	X
15	F	18	1097	12.78	2.68	16.50	8.13	13.03	12.63	30.6	29.8	14.9	6.0	8.9	
16	F	27	160.9	11.3	5.73	11.3	7.71	7.59	3.67	30.6	27.7	9.5	6.3	3.2	X
17	M	22	85.8	0.97	0.48	5.38	4.19	0.00	0.00	30.6	30.3	3.4	2.3	1.1	
18	M	31	213.6	5.61	5.84	10.66	8.08	0.00	0.00	30.6	30.6	9.3	6.3	3.0	X
19	F	30	28.6	0.16	0.10	1.58	1.08	0.35	0.08	30.6	30.6	0.8	0.5	0.3	X
20	F	43	197.5	8.24	7.52	11.40	7.61	8.65	3.54	30.6	30.6	8.2	5.0	3.2	X
21	M	52	777.6	19.8	1.25	16.3	8.55	0.00	0.00	30.6	20.4	15.5	8.1	7.4	
22	M	16	584.4	18.3	0.94	13.4	8.00	0.00	0.00	30.6	21.4	17.2	9.4	7.8	
23	M	17	277.1	11.7	0.46	10.9	5.67	0.00	0.00	30.6	23.2	12.3	5.7	6.6	
24	M	36	325.2	14.79	13.01	13.92	10.46	0.00	0.00	30.6	30.6	16.0	10.5	5.5	X
25	M	44	227.1	13.2	1.88	13.9	8.66	0.00	0.00	30.6	23.8	12.2	8.1	4.1	
26	M	22	415.7	14.0	0.42	13.0	5.5	0.00	0.00	30.6	20.9	12.8	8.3	4.5	
27	F	40	117	6.54	8.13	8.96	8.45	0.88	0.31	30.6	30.6	6.3	5.2	1.1	X
28	F	51	96.1	3.09	0.52	7.60	4.57	3.84	0.50	30.6	30.6	4.1	3.1	1.0	X
29	M	38	330.4	11.6	2.12	12.7	8.02	0.00	0.00	30.6	25.5	11.3	7.0	4.3	
30	F	17	138.2	10.15	0.26	9.74	3.96	2.58	0.08	30.6	23.3	8.3	5.3	3.0	
31	M	35	1559	16.1	0.41	19.1	7.38	0.00	0.00	30.6	17.0	20.3	11.1	9.2	
32	F	76	136.4	7.06	6.16	10.71	10.11	1.30	0.44	30.6	30.6	0.0	0.0	0.0	X
33	M	64	219.1	1.44	0.69	8.81	5.25	0.00	0.00	30.6	30.6	3.3	1.5	1.8	X
34	F	18	113.1	1.67	0.73	5.64	3.37	0.50	0.13	30.6	30.6	3.2	1.6	1.6	X
**median**		**33.5**	**167.6**	**6.79**	**1.51**	**9.79**	**6.19**	**0.10**	**0.02**	**30.6**	**30.6**	**7.6**	**4.9**	**3.0**	
**stdev**		**16.3**	**324.6**	**5.94**	**2.99**	**4.38**	**2.48**	**3.69**	**2.65**	**0.0**	**4.2**	**5.7**	**3.2**	**2.7**	

Note that 0% risk values in Tables S5 and S6 resulted from two patients who were 76 years old at treatment and were beyond the age at which the risk of late effects would contribute to p_{tot}.

Table S7. Dosimetric and risk comparison between the O-OPT 3DCRT plans for CRF=0 with gEUD parameter of 1 and -22 for the 9 patients who were found to have a benefit >1% when O-OPT 3DCRT plans were compared to clinical 3DCRT plans in this study (Table S1a).

Patient Info				O-OPT Heart D_{mean} (Gy)		O-OPT Lung D_{mean} (Gy)		O-OPT Breast D_{mean} (Gy)		O-OPT CTV D_{mean} (Gy)		O-OPT Total Risk (%)		CLN Total Risk (%)
ID	sex	age	CTV (cc)	gEUDp = -22	gEUDp = 1	gEUDp = -22	gEUDp = 1	gEUDp = -22	gEUDp = 1	gEUDp = -22	gEUDp = 1	gEUDp = -22	gEUDp = 1	
3	M	32	561.4	13.62	0.54	12.71	5.79	0.00	0.00	27.16	18.51	13.2	9.1	15.7
8	F	22	161.2	8.42	0.65	9.27	5.26	10.2	2.39	30.60	28.79	7.2	4.8	7.2
21	M	52	777.6	16.32	1.25	14.41	8.55	0.00	0.00	29.32	20.38	13.1	8.1	16.3
22	M	16	584.4	13.13	0.94	10.42	8.00	0.00	0.00	28.27	21.42	12.9	9.4	14.7
23	M	17	277.1	12.65	0.46	8.10	5.67	0.00	0.00	30.60	23.24	9.5	5.7	9.5
25	M	44	227.1	13.61	1.88	12.87	8.66	0.00	0.00	30.60	23.8	11.5	8.1	11.5
26	M	22	415.7	12.96	0.42	10.31	5.5	0.00	0.00	30.60	20.93	11.0	8.3	11.0
29	M	38	330.4	10.46	2.12	10.12	8.02	0.00	0.00	30.60	25.52	9.2	7.0	9.2
31	M	35	1559	20.27	0.41	19.18	7.38	0.00	0.00	30.60	17.00	17.7	11.1	17.7
